# Everolimus-Associated Thrombotic Microangiopathy Following Renal Transplant: A Case Report

**DOI:** 10.7759/cureus.71535

**Published:** 2024-10-15

**Authors:** Zachary Chi Wai Leong, Jason Henn Leong Kong, See Yee Khor, Yew Fong Liew

**Affiliations:** 1 Nephrology, Hospital Pulau Pinang, George Town, MYS; 2 Pathology, Hospital Seberang Jaya, Permatang Pauh, MYS

**Keywords:** microangiopathic haemolytic anaemia, mtor inhibitors, post-renal transplant, renal allograft recipient, thrombotic microangiopathy (tma)

## Abstract

Thrombotic microangiopathy (TMA) is a serious complication that may affect post-renal transplant recipients. De novo TMA has been linked to the use of transplant immunosuppressive agents, including calcineurin inhibitors (CNI) and mammalian target of rapamycin inhibitors (mTORi). We report a case of a 41-year-old female renal transplant recipient who presented with hemolytic anemia, thrombocytopenia, and acute allograft dysfunction. Before her presentation, she was on immunosuppression with oral tacrolimus, oral prednisolone, and oral everolimus. Her renal biopsy showed features of TMA, which led to extensive workup to identify the underlying cause. Eventually, everolimus was recognized as the cause of secondary TMA as her hemolytic parameters and renal allograft function recovered following discontinuation of this drug. This case report highlights the association of everolimus with TMA in a post-renal transplant patient. Early recognition and drug withdrawal can prevent allograft loss.

## Introduction

Thrombotic microangiopathy (TMA) is a rare but serious condition affecting 0.8-14% of renal transplant recipients [1]. It can occur as a de novo disease due to various pathogenic mechanisms or recurrence of a previously known or undiagnosed atypical hemolytic uremic syndrome (aHUS). Hallmark presentation of TMA includes microangiopathic hemolytic anemia, thrombocytopenia, and acute organ dysfunction. Post-transplant immunosuppression with calcineurin inhibitors (CNI) and mammalian target of rapamycin inhibitors (mTORi) have been linked with the development of TMA. Among the mTORi, the occurrence of drug-induced TMA is more frequently seen with sirolimus than with everolimus [2]. This is a rare case of a middle-aged post-renal transplant female patient who presented with acute allograft dysfunction and hematological features of TMA. Extensive investigations and assessment of response to treatment eventually identified everolimus as the culprit drug.

## Case presentation

We present the case of a 41-year-old lady diagnosed with end-stage renal disease. Her primary disease was unknown. She was commenced on peritoneal dialysis for four years before converting to hemodialysis. In 2022, she received an ABO-compatible spousal renal transplant. Immunological risk prior to transplant was high due to the history of four previous pregnancies, the presence of six human leucocyte antigen (HLA) mismatches, and positive donor-specific antibodies (DSA). Pre-transplant induction therapy included thymoglobulin, plasma exchange, and intravenous immunoglobulin (IVIG). Post transplant, she was on immunosuppression with oral mycophenolate mofetil (MMF) 1 g twice a day (BD), oral tacrolimus 3.5 mg BD, and oral prednisolone 5 mg once a day (OD). She had moderate Cytomegalovirus (CMV) risk and developed CMV viremia on day 38 post renal transplant. A CMV DNA level of 1805 IU/ml was detected by quantitative real-time polymerase chain reaction (qPCR). The dose reduction of MMF did not suppress her CMV viremia; subsequently, MMF was replaced with oral everolimus 1.5/2 mg BD. Following conversion to everolimus, repeated CMV polymerase chain reaction (PCR) levels gradually decreased and became undetectable on day 75 post transplant.

Her post-transplant renal profile was stable with a baseline creatinine of 78 to 84 umol/L. Other blood parameters, including full blood counts, were normal during her regular follow-up. During routine blood taking at 18 months post transplant, we noted that her serum creatinine level increased to 174 umol/L. Therapeutic drug monitoring of tacrolimus level was 5.0 ng/mL (target range: 3-5 ng/ml), and everolimus level was 10.5 ng/mL (target range: 3-8 ng/ml). Her mid-stream urine culture grew beta-hemolytic group B Streptococcus sensitive to penicillin group antibiotics. On further questioning, she complained of fever for five days, chills and rigors, nausea, and loss of appetite. Afterwards, she was hospitalized due to allograft dysfunction secondary to a urinary tract infection and she received intravenous amoxicillin-clavulanate (Augmentin) 1.2 g BD (renal dose adjusted to creatinine clearance of 18 ml/min). Everolimus dose was reduced. In the ward, she remained afebrile. Ultrasound of the renal allograft reported no abnormalities.

Serial blood investigations showed new onset progressive anemia and thrombocytopenia on day four of admission. An urgent peripheral blood film showed significant red cell fragmentation with 6% schistocytes suspicious of TMA. Reticulocyte count was raised at 5.2% and lactate dehydrogenase (LDH) was 1203 iu/L but the total bilirubin level was normal at 16 umol/L. Her Coombs test was negative and her coagulation profile was within normal limits, with an international normalized ratio (INR) of 1.12 and activated partial thromboplastin time (APTT) of 27.2 seconds. A blood test to evaluate for ADAMTS13 activity was sent urgently to exclude thrombotic thrombocytopenic purpura (TTP) but results were not immediately available. Empirical treatment for TTP with therapeutic plasma exchange (TPE) was started on day five of admission. On day six, IV methylprednisolone 500 mg daily for three days was added due to a lack of improvement in serum creatinine and hemolytic parameters.

We investigated for other possible causes of post-transplant TMA, including infection and drug-induced and acute allograft rejection. CMV and BK virus PCR were undetected. The repeated everolimus drug level following dose reduction was 5.3 ng/ml. DSA were sent but were pending results. Renal biopsy was delayed due to concerns of bleeding risk with a low platelet count of 48 x 10^3^/uL. On day eight of admission, after four cycles of TPE and completion of IV methylprednisolone, we observed a mild improvement in serum creatinine (137 umol/L) and platelet count (88 x 10^3^/uL) but repeated peripheral blood film showed increased red blood cell fragmentation with 8% schistocytes. Due to a lack of response to initial therapy, drug-induced TMA was considered and tacrolimus was withheld.

A renal biopsy was performed on day nine of admission. Her pre-biopsy platelet count was 85 x 10^3^/uL. She received two units of platelets due to the risk of post-renal biopsy bleeding associated with thrombocytopenia. The patient was continued on another four cycles of TPE. On day 12 of admission, repeated peripheral blood film showed persistent thrombocytopenia and RBC fragmentation despite withholding tacrolimus for four days. Tacrolimus was restarted and everolimus was withheld. Marked improvement in platelet count, serum creatinine, and LDH was observed after stopping everolimus. TPE was discontinued on day 13 when her platelet count increased to 105 x 10^3^/ul and her platelet count normalized on day 15.

**Figure 1:**
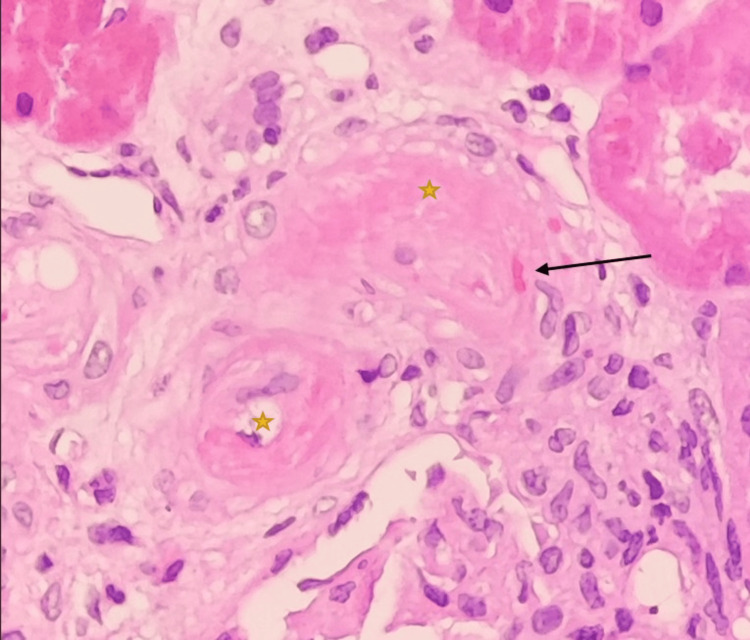
Hematoxylin and eosin (H&E) stain shows one arteriole with marked intimal thickening and near total luminal occlusion (yellow star). Also, there are entrapment and fragmentation of red blood cells (black arrow) within the arteriolar wall (H&E stain, under 400x magnification).

**Figure 2:**
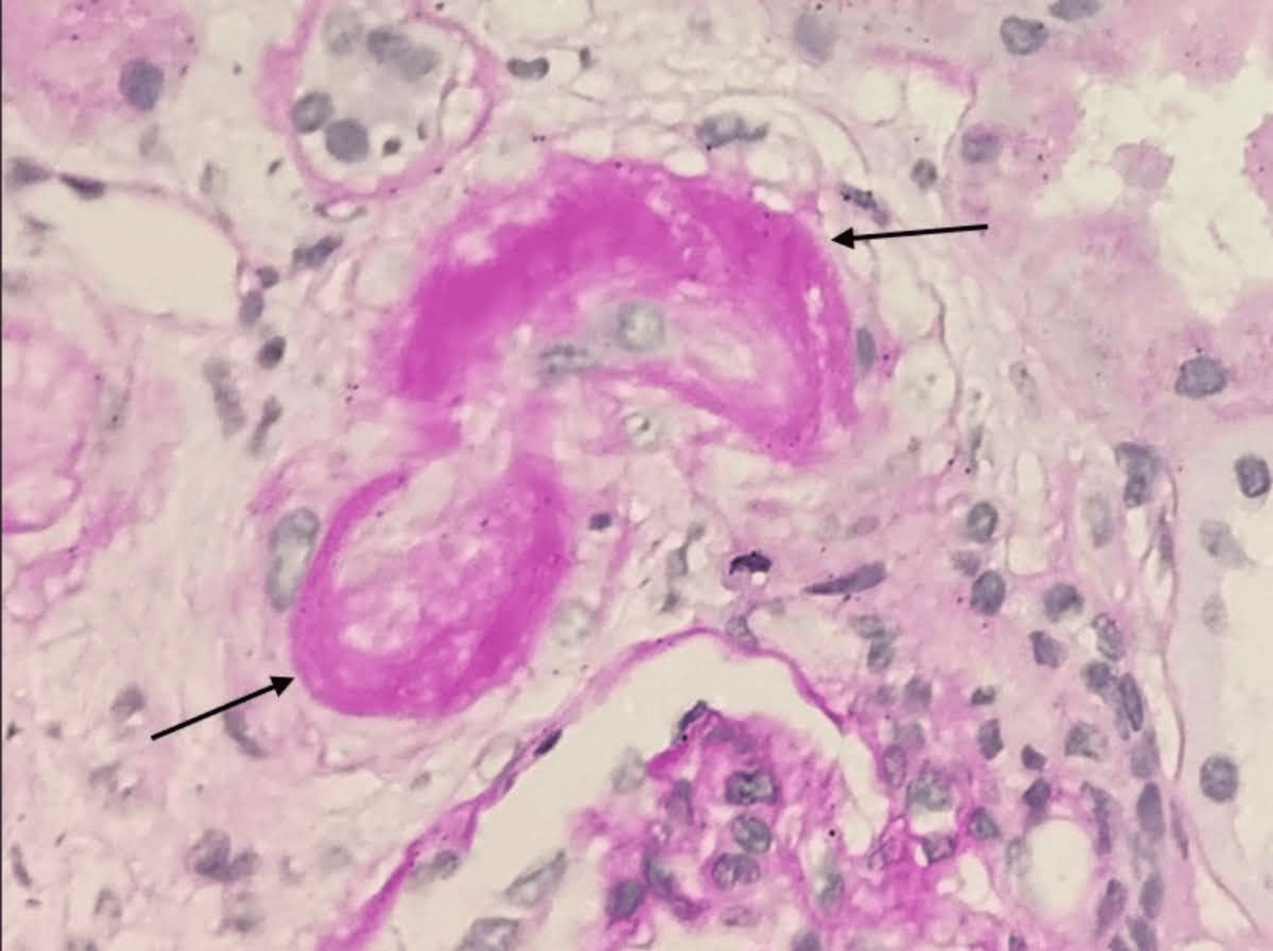
Periodic-acid Schiff (PAS) stain highlights the mucoid intimal thickening of the arteriolar wall and near total luminal occlusion (PAS stain, under 400x magnification).

Renal allograft biopsy showed tubulitis and mild to moderate interstitial inflammation involving 30% of the non-scarred area of the cortex in keeping with acute T cell-mediated rejection (TCMR), Banff category 4, grade 1A. One arteriole showed marked thickening of the intima with almost complete luminal occlusion and the presence of fragmented red blood cells within the arterial wall. which are features of TMA (Figures 1, 2). There was no histological evidence of antibody-mediated rejection.

**Figure 3:**
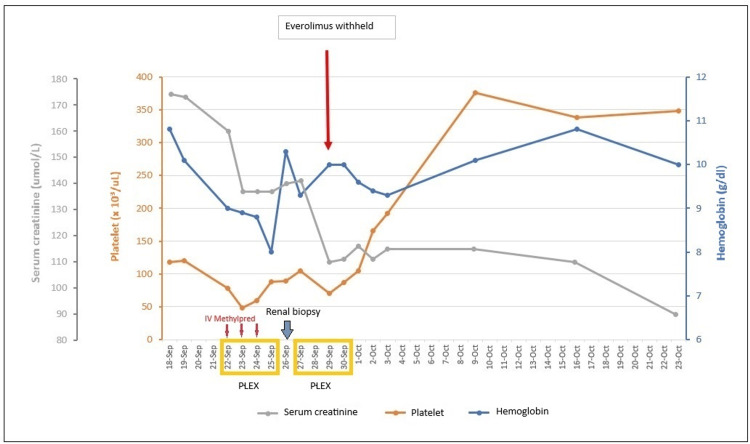
Hemoglobin, platelet, and serum creatinine trends and response to IV methylprednisolone, plasma exchange, and everolimus withdrawal.

The patient was discharged on day 16 with hemoglobin (Hb) at 9.3 g/dL, platelet count of 192 x 10^3^/uL, and serum creatinine at 115 umol/L (Figure 3). Her immunosuppressive therapy on discharge was oral tacrolimus 2.5/2 mg BD, oral MMF 250 mg BD, and oral prednisolone 5 mg OD. Follow-up blood investigations three weeks post discharge showed a hemoglobin level of 10 g/dL, platelet count of 348 x 10^3^/uL, and serum creatinine of 90 umol/L. DSA reviewed during follow-up were negative and ADAMTS13 activity was normal (91%). One year later, her serum creatinine remained stable at 99 umol/L and CMV PCR remained undetected.

## Discussion

TMA is a rare condition characterized by microvascular occlusion by fibrin clots precipitated by endothelial injury. De novo TMA is rare in post-renal transplant recipients but may cause severe dysfunction and loss of allograft if not diagnosed and treated promptly [3]. Risk factors associated with the development of post-transplant de novo TMA include antibody-mediated rejection (ABMR), viral infections (CMV, BK virus, parvovirus B19), ischemic-reperfusion injury, immunosuppressive drugs (calcineurin inhibitors, mTOR inhibitors), ABO-incompatible transplant, and marginal kidneys [4-6]. Clinical onset of de novo TMA generally begins in the early post-transplant period when higher immunosuppression doses are used. However, there are also reported cases of late-onset TMA up to two to six years post transplant [7]. Early onset post-transplant TMA is linked to higher rates of graft loss.

As seen in our patient, TMA typically presents with a triad of hemolytic anemia, thrombocytopenia, and evidence of organ impairment (acute allograft dysfunction) due to microcirculatory disturbance. Laboratory markers of hemolysis include elevated indirect bilirubin, LDH, reticulocyte count, and increased percentage of schistocytes on peripheral blood film (≥1%). In the setting of TMA, the Coombs test is negative and the coagulation profile is within normal limits. When clinicopathologic evidence of TMA is present, it is important to investigate the underlying etiology to offer therapy directed toward the causal pathogenic process.

TTP is a hematological emergency with TMA presentation. This condition develops in the setting of severe ADAMTS13 activity deficiency (<10%). Although TTP does not usually present with severe acute kidney injury or renal-limited TMA, it is crucial to recognize this condition early as it is associated with high mortality (90%) if left untreated [8]. Measurement of ADAMTS13 activity is helpful to confirm or exclude this diagnosis but is often limited by delayed test results. Early treatment with TPE can reduce mortality to 20% [9], hence many treating physicians have a low threshold to initiate TPE for TTP even before the test results are available. Similarly, we initiated TPE promptly for our patient.

Given our patient’s high immunological risk profile, acute allograft rejection was considered. Renal biopsy remains the gold standard for diagnosing acute allograft rejection. In this case, we had to delay renal allograft biopsy due to low platelet counts. A renal biopsy was only performed on day nine of admission and her pre-biopsy platelet count was 85 x 10^3^/uL. Although platelet transfusion in TMA carries the risk of arterial thrombosis, she was transfused with two units of platelets due to concerns about post-renal biopsy bleeding complications associated with her low platelet count. The risk for post-renal biopsy bleeding is incrementally increased when platelet levels decrease to <200 × 10^3^/uL and more significant bleeding occurs when platelets decrease to <120 × 10^3^/uL [10]. Renal histopathological findings were Banff grade 1A TCMR, acute tubular injury, and TMA. Petr et al. reported associations between TMA and antibody and T cell-mediated rejection [11]. In their study, T cell-mediated rejection occurred in 11 out of 93 cases of renal biopsy-proven TMA.

There was some improvement in our patient’s serum creatinine from 166 to 137 umol/L, which may reflect the response of acute TCMR to pulsed IV methylprednisolone. However, serial peripheral blood films showed no improvement in hemolysis parameters and platelet counts. A trial of withholding tacrolimus for four days did not result in any improvement. Fortunately, her hemolytic parameters and platelet count improved and normalized after everolimus withdrawal.

Calcineurin inhibitors (cyclosporin A, tacrolimus) and mTORi (everolimus, sirolimus) have been reported to cause transplant-related TMA [12]. Three mechanisms that contribute to the development of CNI-induced TMA are as follows: (1) increased synthesis of vasoconstrictor agents such as endothelin and thromboxane A2, and decreased vasodilator nitric oxide and prostaglandin; (2) increased platelet aggregation and antifibrinolytic activity; and (3) alternative complement pathway activation by microparticles produced by endothelial cells [13]. The risk of TMA is reportedly higher with cyclosporine than with tacrolimus.

Several studies have documented an increased risk of TMA with combined CNI and mTORi. Fortin et al. reported that patients on combined therapy have the highest risk of developing de novo TMA [14]. An explanation for this increased risk is that mTORi impair the repair of endothelial cell injury induced by CNI. In a study involving 396 renal transplant recipients, 36 (7.3%) developed TMA, and 17 were drug-related [15]. It was found that not only individual drug levels of CNI and mTORi were higher but also the sum of both drug levels. Combined monitoring of both drugs was suggested to prevent this complication [16].

Management of drug-induced post-transplant TMA involves dose reduction or stopping the offending agent and switching to an alternative. In CNI-induced TMA, options are to switch to a different CNI or to mTORi. The effectiveness of this strategy is controversial. Kwon et al. reported that switching CNI resulted in a good one-year graft function response in 81% of post-transplant TMA patients [17]. Zarifian et al. showed that despite observed improvement in 81% of patients who switched to another CNI, 30.1% lost renal allograft function due to TMA [5]. Satoskar et al. showed no difference in outcomes regardless of stopping or switching immunosuppression [18].

Plasma exchange has been utilized along with the discontinuation of CNIs. Withdrawal of CNI therapy alone may not be sufficient to reverse effects due to the activation of an alternate complement pathway [13]. Postulated benefits of plasma exchange are the removal of platelet aggregation factors such as thromboxane A2 and replenishment of deficient factors, e.g., prostaglandin I2 stimulating factor. In a case series of 29 patients with post-transplant TMA who were treated with plasma exchange and discontinuation of CNIs, 80% (23/28 patients) recovered graft function [19]. The optimal duration of plasma exchange and therapeutic endpoints remain unknown.

## Conclusions

In conclusion, our case report highlights the occurrence of everolimus-induced TMA in a post-renal transplant patient. Given the growing use of mTORi in transplant immunosuppression with newer CNI minimization strategies, clinicians should be aware of this rare but serious complication. Post-renal transplant patients presenting with microangiopathic hemolytic anemia, thrombocytopenia, and acute allograft dysfunction while on immunosuppression with CNI or mTORi should prompt consideration of drug-induced TMA. Early recognition and discontinuation of these drugs are essential to prevent allograft loss.
